# Special Issue “Delivery Systems of Peptides and Proteins: Challenges, Status Quo and Future Perspectives”

**DOI:** 10.3390/ph16091285

**Published:** 2023-09-12

**Authors:** Wenjuan Liu, Haisheng He, Jianping Qi

**Affiliations:** Key Laboratory of Smart Drug Delivery of MOE, School of Pharmacy, Fudan University, Shanghai 201203, China; 21111030046@m.fudan.edu.cn

Peptides and proteins have emerged as more important therapeutic molecules compared to small molecular chemicals due to their high specificity and efficacy and low toxicity. However, the intrinsic properties of peptides and proteins, including their large molecular weight, poor chemical and conformational stability, short half-life and low permeability, pose tremendous challenges to clinical use, as well as to researchers of medical and pharmaceutical science [1,2]. In order to fully explore the potential of peptides and proteins for the treatment of severe diseases, various injectable and non-injectable administration routes have been utilized, such as nasal, ocular, pulmonary and transdermal delivery [3,4,5]. An increasing number of novel delivery systems such as exosomes, ionic-liquid-assisted nanoparticles and polymeric micelles have been developed to improve the efficacy of peptides and proteins [6,7,8,9]. This Special Issue collects six articles that present the insights of scientists in the field of delivery systems of peptides and proteins.

Although peptides and proteins are difficult to be orally absorbed into blood circulation, some oral peptides can play a critical role in the treatment of local gastrointestinal tract diseases [10]. The second article in this Special Issue investigated the interchangeability of two encapsulated pancreatin formulations which are indicated in the treatment of exocrine pancreatic insufficiency, i.e., Kreon^®^ (Creon^®^, Polfa Warszawa S.A., Warszawa, Poland) and Lipancrea^®^ (Mylan Healthcare. Warszawa, Poland, part of Viatris group, Canonsburg, PA, USA). In order to ensure their efficacy in the harsh gastrointestinal environment, various formulation approaches are always required to protect against degradation. In other words, their clinical efficacies highly depend on protection by formulations [11].

In addition, the blood–brain barrier (BBB) lies between blood circulation and the brain, impeding the transport of peptides and protein into the brain. Yue and colleagues gave a systematic review of localized delivery strategies for peptide/protein therapeutics in the treatment of central nervous systems (CNS) diseases, such as intracerebroventricular, intraparenchymal convection-enhanced, intrathecal and intranasal administration. Local delivery strategies could circumvent the blood–brain barrier to increase protein/peptide concentration. On the other hand, it is noteworthy that complications after surgical implantation and potential foreign repulsion responses could occur [12].

To efficiently deliver proteins and peptides, the formulation approaches and penetration enhancers are always the most critical factors. Cui et al. reviewed recent advancements in oral liposomes for the delivery of peptides or proteins. Considering the vulnerability of liposomes along the digestive tract, improvements in the components of liposomes or surface modification were developed. The authors envisaged that deeper understanding of the processes involved in the oral absorption of liposomes could contribute to designing more efficient delivery systems and thus improving the bioavailability of oral polypeptides or proteins [13]. In addition to injectable administration, the transdermal drug delivery of biomacromolecules has also attracted extensive attention. Zhang et al. summarized novel penetration enhancers, nano vesicles and microneedles as efficient strategies to overcome stratum corneum and the tight junctions between keratinocytes for both localized and systemic treatment. It was necessary to elucidate the latent mechanism of different strategies and associated side effects [14].

Pulmonary administration, another alternative to the injectable route, can not only accelerate the absorption rate of peptide drugs, but also improve bioavailability. Huang et al. provided several perspectives regarding the design of metered-dose inhalers (MDIs) to solve the bottleneck of low-peptide drug loading [15].

Having underlined the significant role of delivery systems of peptides and proteins, this Special Issue covers early development and later industrialization. Questions remain about the manufacturability, cost and toxicology of delivery systems. The rapid advancements in protein chemistry, formulation and drug delivery are anticipated to accelerate the emergence of superior delivery systems of peptides and proteins. With thorough consideration, versatile and efficient drug delivery systems will progress toward clinical trials or even clinics. Finally, the Guest Editors would like to sincerely thank all the authors and reviewers for their valuable contributions. We would also like to thank MDPI for deciding to publish this Special Issue.
